# Eco-friendly nanocomposites derived from geranium oil and zinc oxide in one step approach

**DOI:** 10.1038/s41598-019-42211-z

**Published:** 2019-04-12

**Authors:** Ahmed Al-Jumaili, Peter Mulvey, Avishek Kumar, Karthika Prasad, Kateryna Bazaka, Jeffrey Warner, Mohan V. Jacob

**Affiliations:** 1 Electronics Materials Lab, College of Science and Engineering, James Cook University, Townsville, QLD 4811 Australia; 2 Physics Department, College of Science, Ramadi, Anbar University, Ramadi, 11 Iraq; 3 Australian Institute of Tropical Health and Medicine, James Cook University, Townsville, QLD 4811 Australia; 4 School of Chemistry, Physics, Mechanical Engineering, Queensland University of Technology, Brisbane, QLD 4000 Australia

## Abstract

Nanocomposites offer attractive and cost-effective thin layers with superior properties for antimicrobial, drug delivery and microelectronic applications. This work reports single-step plasma-enabled synthesis of polymer/zinc nanocomposite thin films via co-deposition of renewable geranium essential oil-derived polymer and zinc nanoparticles produced by thermal decomposition of zinc acetylacetonate. The chemical composition, surfaces characteristics and antimicrobial performance of the designed nanocomposite were systematically investigated. XPS survey proved the presence of ZnO in the matrix of formed polymers at 10 W and 50 W. SEM images verified that the average size of a ZnO nanoparticle slightly increased with an increase in the power of deposition, from approximately 60 nm at 10 W to approximately 80 nm at 50 W. Confocal scanning laser microscopy images showed that viability of *S. aureus* and *E.coli* cells significantly reduced on surfaces of ZnO/polymer composites compared to pristine polymers. SEM observations further demonstrated that bacterial cells incubated on Zn/Ge 10 W and Zn/Ge 50 W had deteriorated cell walls, compared to pristine polymers and glass control. The release of ZnO nanoparticles from the composite thin films was confirmed using ICP measurements, and can be further controlled by coating the film with a thin polymeric layer. These eco-friendly nanocomposite films could be employed as encapsulation coatings to protect relevant surfaces of medical devices from microbial adhesion and colonization.

## Introduction

There has been an increased interest in the functionalizing of sustainable resources-derived polymers via incorporation of metallic nanoparticles, where the intrinsic properties of the nanoparticles are contributed into the polymer^1^. The resultant ‘eco-friendly composites’ combine the advantages of low-dimensional organic layers with an enormous surface area of nanoparticles, creating a wide range of promising applications in science and manufacturing^2^. These composites are versatile, potentially biodegradable, and their polymer can be derived from a wide variety of possible renewable precursors, such as oxygen-rich monomers and hydrocarbon-rich monomers^3,4^. Intelligent use of eco-friendly nanocomposites have the potential to reduce the growing impact of modern day technology on ecosystems (e.g. pollution and waste disposal) while sustaining the development of nanotech-driven applications.

The potential to modify chemical, physical and/or bio-responsive properties of solid surfaces (e.g. medical devices and implants) without affecting their bulk properties is the key utilization of composite thin films in electronics, biomaterials, and other relevant industries^5,6^. In particular, nanocomposites could be effectively employed as antibacterial coatings, where the metal nanoparticles are incorporated into a thin layer of polymer, providing a high platform for active particles to interact with microorganisms. The main benefit of these composites is the extremely low quantity of selected nanoparticles required to achieve desired outcomes due to powerful antimicrobial properties of metal nanoparticles^7^.

Plasma polymerization is one of the rapidly advancing techniques for deposition of smooth, uniform, organic thin films from naturally-available alternatives (e.g. essential oils and herb extracts) on different substrates^8^. Essential oil-based coatings prepared by plasma polymerization display a wide range of desired properties, including biocompatibility, optical transparency^9,10^, and moderate hydrophilicity^11^. These films have found a host of potential applications in biomaterials (e.g. as biocompatible and antimicrobial surfaces) and electronics (e.g. as layers in superior organic and hybrid devices)^12,13^.

By introducing inorganic particles into the plasma polymer matrix, it may be possible to further enhance the properties of plasma polymers. Plasma systems can be adapted to introduce inorganic particles in the structure of the polymer matrix as it is formed^14,15^, where the chemical reaction in the gas-phase and the nucleation/growth of nanoparticles happen simultaneously. Consequently, plasma-formed composite materials comprise inorganic particles and their clusters trapped within a highly cross-linked polymer matrix consisting of short polymeric chains that are randomly branched and terminated. While the properties of polymer-metal composites fabricated using simultaneous plasma polymerization and metal evaporation have been reported, they typically use conventional monomers^16,17^. To the best of our knowledge, there is no systematic study investigating nanocomposite plasma films derived from essential oils and inorganic nanoparticles.

Among various metals, zinc oxide nanoparticles have an attractive set of properties that include a powerful antibacterial performance against a variety of pathogenic microorganisms, high luminous transmittance chemical/physical stability, and excellent catalytic activity^18–20^. Furthermore, zinc oxide is relatively low cost, available in commercial quantities and can take several morphological forms (e.g. spherical particles, nano-rode, etc). While there are various physical/chemical methods to produce nano-sized zinc oxide, thermal decomposition of zinc acetylacetonate (Zn(acac)_2_) is known to generate zinc oxide nanoparticles of different sizes and morphologies^21–24^. This process can be integrated with a plasma polymerization system to enable *in situ* functionalization of the polymer (during chains formation) in the absence of a catalyst. Also, it allows for a wide variety of metals and organic precursors to be combined, and guarantees a minimum contamination rate.

This paper reports the fabrication and characterizations of nanocomposite films produced using a single-step approach that combines simultaneous plasma polymerization of renewable geranium essential oil with thermal decomposition of Zn(acac)_2_. To the best of our knowledge, this is the first study on the synergy between geranium oil and ZnO nanoparticles in an eco-friendly coating context.

## Materials and Methods

### Precursors

Geranium essential oil was nominated because it is highly volatile at room temperature, where no external heating system or carrier gases are required to transport the precursor molecules to the spot of fabrication. Moreover, geranium oil possesses strong antibacterial activity toward gram-negative and gram-positive bacteria, which under certain polymerization conditions can be retained in the fabricated films.

Geranium essential oil was obtained from Australian Botanical Products (ABP, Victoria, Australia). As stated by the manufacturer, this geranium oil is extracted through a steam distillation process from leaves of *Pelargonium graveolens*. The geranium oil contains citronellol (32%), geraniol (15%), linalool (6%), isomenthone (6%), geranyl formate (2.5%), tiglate (2%), citronellyl formate (6%), guaia-6,9-diene, and 10-epi-γ eudesmol (5%), as well as trace amounts of other secondary plant metabolites. Geranium oil was used without further modification in all experiments.

Zinc acetylacetonate hydrate powder obtained from Sigma-Aldrich (Germany) was used (without further purification) as the zinc precursor. Zn(acac)_2_ was selected due to its relatively low decomposition temperature, which made it possible to integrate gas-phase catalyst-free nanoparticle nucleation and synthesis with plasma polymerisation.

### Thin films synthesis

Microscope glass slides (76 mm × 26 mm) and round cover-glass sheets (*d* = 19 mm) were sonicated in a solution of water and commercial decon for 20 min, then washed with distilled water, rinsed in acetone and propan-2-ol, and finally dried using an air gun. Plasma polymerization was carried out in a cylindrical glass tube (*l*: 80 cm, *d*: 5 cm) fitted with two external parallel copper rings, which were used as electrodes connected to a generator through a matching network. A radio frequency generator model ACG-3B (MKS Instruments, Andover, MA, USA) was operated in a continuous wave mode (13.56 MHz) to create a glow discharge. The distance between electrodes and the distance between monomer and the electrodes were optimized to achieve the optimum plasma stability. Prior to each fabrication, the reactor tube was evacuated to a pressure of 0.2 mbar utilizing a double stage rotary pump (JVAC–DD150, Victoria, Australia). Furthermore, the monomer flow rate (*F*) was estimated using the relation 1, derived from the ideal gas formula^25^:

1 $$F=\frac{dp}{dt}\times 16172\frac{V}{T}$$

where *p* is the pressure inside the plasma tube (mbar), *t* is time (s), *V* is the volume of the plasma tube (L), and *T* is the ambient temperature (K). Initially, the tube was evacuated to 0.2 mbar, then the monomer gas was released into the tube until the pressure reached a stable value, at which point the outlet valve was closed, and the pressure was measured every 5 s for 1 min. The geranium flow rate was calculated to be 16.22 cm^3^/min.

The input radio frequency powers used for the deposition of pristine polymers were 10 W and 50 W for 20 min, with the resulting films abbreviated as Ge 10 W and Ge 50 W, respectively.

**Figure 1:**
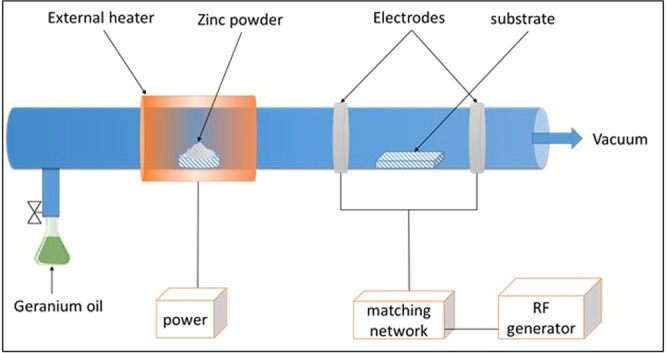
Plasma polymerization system equipped with an external heater for thermal decomposition of metallic powders.

In the case of composite fabrication, the plasma polymerization tube was supplemented with an external ceramic fiber heater, where Zn(acac)_2_ powder (0.05 g) was placed in a ceramic boat inside the plasma tube and heated to 200 °C, as seen in Fig. 1. The Zn(acac)_2_ vapor was carried into the fabrication zone (i.e. where substrates were located) by the flow of geranium vapors in the direction from the flask to the pump outlet. ZnO nanoparticles were formed in the gas phase and imbedded into the polymer matrix during the growth of the latter. The input power used for the deposition of all composites were 10 W and 50 W for 20 min, and the resulting composite structures were abbreviated as Zn/Ge 10 W and Zn/Ge 50 W, respectively.

The coating thickness of pristine and composites films were estimated to be around 500 nm. As the thickness of composites may vary slightly due to the presence of nanoparticles on the film surface, in order to investigate the release of zinc from the composites, layers of 25 nm and 50 nm were coated onto the surface of Zn/Ge 10 W and Zn/Ge 50 W. These films were fabricated on glass substrates, and coating thickness was estimated using Variable Angle Spectroscopic Ellipsometry (JA Woollam-M2000 D, Lincoln, NE, USA) following the protocol outlined in our previous work^26^. The thickness was controlled by optimizing the time of deposition, where 60 s and 100 s yielded approximately 25 nm and 50 nm. The flow rate of geranium oil was kept at 16.22 cm^3^/min during the coating process.

## Material Characterization

### Chemical composition

X-ray photoelectron spectroscopy (XPS) spectra were obtained by Specs SAGE 150 spectroscope supplemented with a monochromatic Al Kα X-ray source (*hυ* = 1486.6 eV). The measurements were collected at a take-off angle of 90° from a circular area with a diameter of ~5 mm. Intending to reduce X-ray-induced polymer degradation, the exposure time was adjusted to the minimum required to achieve a sufficient signal-to-noise ratio. Surface charging effects of the samples were addressed by a reference value of 285.0 eV, which is the binding energy of C 1 s peak originating from neutral hydrocarbon (CH_x_). The elements’ concentrations were estimated using Casa XPS software.

### Surface characteristics

Scanning Electron Microscopy (SEM) (SU5000, Hitachi, Canada) with Energy Dispersive X-ray Spectroscopy (EDS) were used to study surface properties of the fabricated films. Films were fabricated on nickel foil, a conductive substrate, to avoid the scattering from glass substrates. Data were acquired at Vacc = 3.0 kV, EC = 115 K nA, WD = 6.1 mm, and a high vacuum atmosphere.

Surface characteristics were also investigated by employing a low-noise scanning/high-resolution atomic force microscope AFM (NT-MDT NTEGRA, Moscow, Russian Federation) in the tapping mode, with a scanning area of 10 µm × 10 µm. For all samples, data were acquired under ambient conditions. Then, the data were analyzed using Nova software (Version 1.0.26, Moscow, Russia), with the fitting correction value (polynomial order of 4).

Static contact angle measurements were recorded to determine the wettability of resultant films. A drop of double distilled water was gently dispensed onto the surface of samples with a micro-syringe^27^. Water drop contact angles were calculated using data collected by means of a goniometer (KSV CAM 101, Helsinki, Finland), where a minimum of five measurements per sample were collected. To reduce inaccuracies in the obtained data, the size of the droplet was carefully selected to be approximately 2.5 µL in volume.

### Bacterial Studies

The antibacterial activity of the pristine and composite films was determined by the Live/Dead staining method using gram-negative *Escherichia coli* and gram-positive *Staphylococcus aureus*. These pathogens are well-known sources of infections in hospitals and implantable devices.

For each experiment, a fresh suspension was prepared by first refreshing the frozen stock culture (1 mL) in Oxoid nutrient broth (10 mL) at 37 °C and shaken at 120 rpm. A spectrometer (The SPECTROstar Nano, BMG labtech, Germany) was employed to calculate cell numbers in a bacterial suspension prior to placement on polymer surfaces. The cell density was adjusted to (OD_600_ = 0.1) to ensure a uniform starting culture (2 × 10^5^ CFU/mL).

The experiment was run in triplicate. Polymer-coated glass slides (*d* = 19 mm) and controls (uncoated glass slides) were placed into 12-well plates, and an aliquot of 2 ml of bacterial suspension at a concentration of 2 × 10^5^ CFU/mL was placed onto the sample surface. All samples were incubated for 24 h at 37 °C and 5% CO_2_.

After incubation, a staining kit (SYTO™ Invitrogen, Thermo Fisher, USA) was used to study live/dead bacterial cells. The dyes were applied following the protocol outlined in^28^. Prior to imaging, bacterial suspensions were removed from the samples. Then, 90 μl of the stain was placed on top of each sample and kept in the dark. After 20 min, samples were gently washed with 2 ml of distilled water to remove unattached cells. Immediately, fluorescent images were obtained with a confocal scanning laser microscope (LSM 800, ZEISS, Germany), where green and red colors were indicative of live and non-viable cells, respectively. Viability was calculated as the percentage of viable, adhering bacteria relative to the total number of attached bacteria to the surface.

All data were acquired with a minimum of three replicates each. The standard error of the mean was calculated to define variance about the mean. Statistical analysis of numerical data was determined using a paired t-test. Statistical significance (*) was considered at p < 0.05. Furthermore, a confidence interval (95%) was given at each time point, and was used as an additional pathway to ensure statistical differences between experimental and control data.

To obtain SEM observations, bacterial cells were incubated on the samples for 24 h at 37 °C. After incubation, bacterial suspensions were gently removed and a volume of 2 ml of phosphate-buffered saline was used to rinse each sample. Then, samples were passed through a series of ethanol solutions in ascending order (30%, 50%, 70%, 80% and 100% ethanol) for 10 min per step to remove water. The dehydrated samples were coated with a thin layer of palladium (<5 nm) using an ion sputter for 80 sec and observed using a SEM (SU5000, Hitachi, Canada) at different magnifications.

### Zinc release in aquatic medium

**Figure 2:**
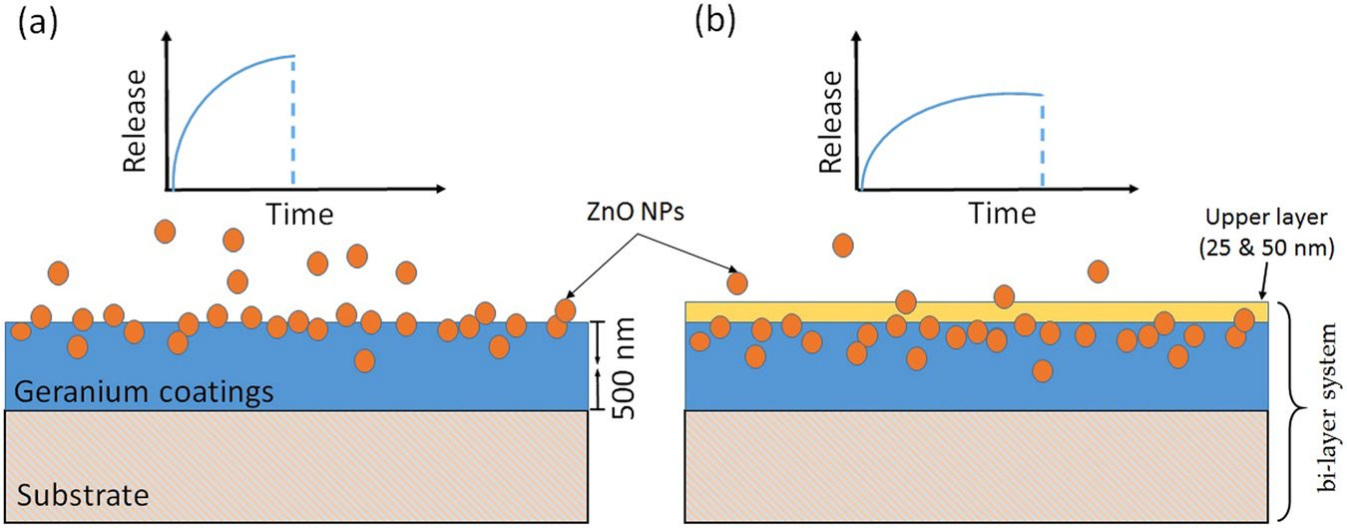
Schematic of the cross-section of (**a**) ZnO/geranium plasma polymer composite films (film thickness is ≈500 nm) with a fast release of ZnO nanoparticles. (**b**) ZnO/geranium films with a thin upper layer of geranium polymer (thickness is ≈25 and 50 nm) with reduced release rate of the ZnO from the composites.

To adjust the zinc release, Zn/Ge composite films were coated by an extra thin layer (25 ± 5 or 50 ± 5 nm) of geranium plasma polymer, as seen in Fig. 2. The releasing rate was measured using inductively coupled plasma-mass spectrometer (ICP-MS 820, Varian, USA). Films were fabricated on glass cover slips and immersed in water. To stay well below saturation, the volume of water added to all samples was set at 5 ml, and incubation took place at 22 °C in the dark. After the deposition of a top polymer coating, samples were incubated in water for 1, 4, 8, 16, 20, 24, 28, 32, 36, and 40 h, and afterwards analyzed by ICP. The calibration was performed using double distilled water as a reference, and zinc concentration < = 5 µg/L.

## Results and Discussion

### Compositional studies

**Figure 3:**
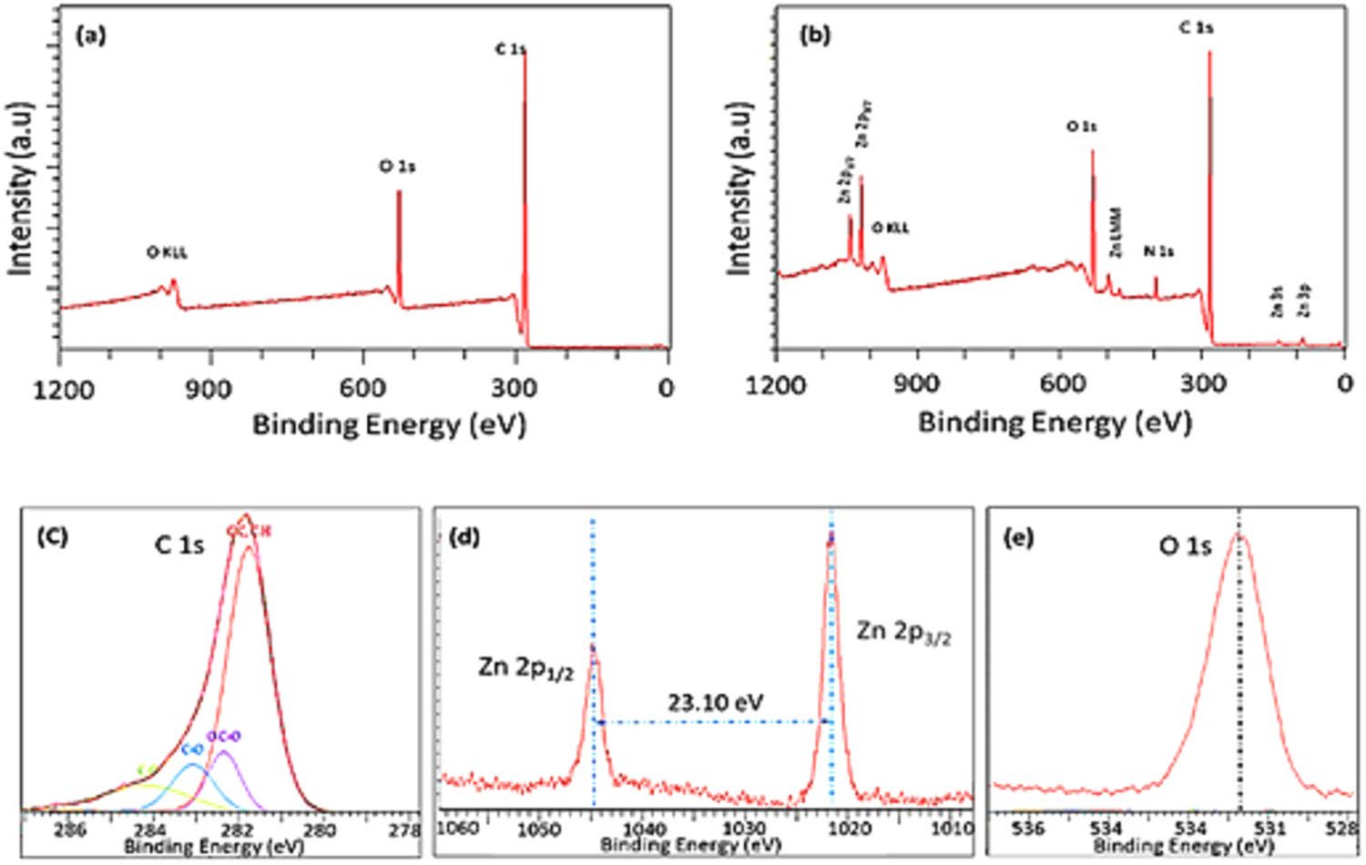
Full scan XPS spectrum of pristine polymer (**a**) and composite (**b**) thin films. The carbon C 1 s binding energy of the pristine polymer is given in (**c**). The 2p symmetrical zinc oxide binding energies are shown in (**d**). O 1 s binding energy for the composite is presented in (**e**).

Full scan XPS spectra of pristine and composite thin films are presented in Fig. 3. The binding energies were calibrated within an accuracy of 0.1 eV using C 1 s (284.6 eV). The obtained peaks were resolved using the CasaXPS software. The spectrum for pristine polymer shown in Fig. 3(a) displays the presence of strong carbon and oxygen at peaks at 282 and 531 eV, respectively. The C 1 s peaks for geranium polymers were fitted with four peaks, which can be ascribed to major hydrocarbon C–C/C–H (BE = 284.9 eV), and other functional groups, such as ether C–O (BE = 286.2 eV), carbonyl C = O (BE = 287.5 eV), and ester O–C = O (BE = 288.9 eV)^29^, as seen in Fig. 3(c).

The spectrum in Fig. 3(b) demonstrates the presence of zinc in the composite structure, along with carbon, oxygen, and nitrogen. No major chemical shifts or asymmetry in the C 1 s and O 1 s peaks could be observed for the composite when compared to pristine polymers. Nevertheless, the presence of 2p (2p_3/2_ and 2p_1/2_) symmetrical zinc oxide binding energies (at 1021.32 and 1044.60 eV) is apparent. Zinc oxide had significantly split spin-orbit components Δ_metal_ = 23 eV, as seen in Fig. 3(d). Furthermore, a minor peak was also observed at 400.08 eV, attributed to nitrogen. This peak most likely originated from impurities within the Zn(acac)_2_ precursor, as noted in previous studies^30^.

Atomic fractions were also calculated and are presented in Table 1. Carbon was identified to be the main element, which contributed up to 85% of the total atomic concentration for samples. Oxygen was identified as the second most abundant element in the films. No impurities were identified for any of the pristine polymers.

**Table 1:** Atomic percentages of pristine and zinc-polymer composite materials fabricated at 10 W and 50 W.

Sample	Atomic percentages (%)			
	C 1 s	O 1 s	N 1 s	Zn 2p
Ge 10 W	85.46	14.45	—	—
Ge 50 W	87.80	12.20	—	—
Zn/Ge 10 W	83.35	13.00	2.87	0.79
Zn/Ge 50 W	78.33	16.86	3.29	1.57

In contrast, the atomic concentrations for the composites were slightly varied. Carbon was identified to be around 80%, while oxygen was above 13.00%. Zinc was shown to be at 0.79% and 1.57% for samples fabricated at 10 W and 50 W, respectively. It is evident that the carbon percentage in the composites decreased with an increase in the power of deposition, which contrasted to the trend observed in the pristine counterparts. The most rational elucidation for this observation is that the higher input power resulted in a higher fragmentation/dissociation of the zinc acetylacetonate, accompanied by the loss of hydrocarbon groups owing to increased ion bombardment, causing a relative increase in oxygen, nitrogen and zinc quantities.

Owing to the nature of deposition in weakly ionized plasmas (where there is high probability of monomers not being ionized), it is possible that geranium will attach to the substrate-surface in its unmodified or minimally modified state, particularly when deposition is performed at low input powers. Those un-fragmented components trapped within the polymer may elute over time, thereby contribute to the ability of the coating to retard microbial attachment on the surface^31^. In addition, un-fragmented geranium (involving aromatic acyclic monoterpene alcohols) may interact with adjacent zinc nanoparticles. For example, formation of zinc oxidants during polymerization can result in oxidation of alcohol moieties to ketones. The presence of long chains of alcohols may marginally increase the size of formed ZnO particles^32–34^.

### Surface characteristics

#### Scanning electron microscopy

**Figure 4:**
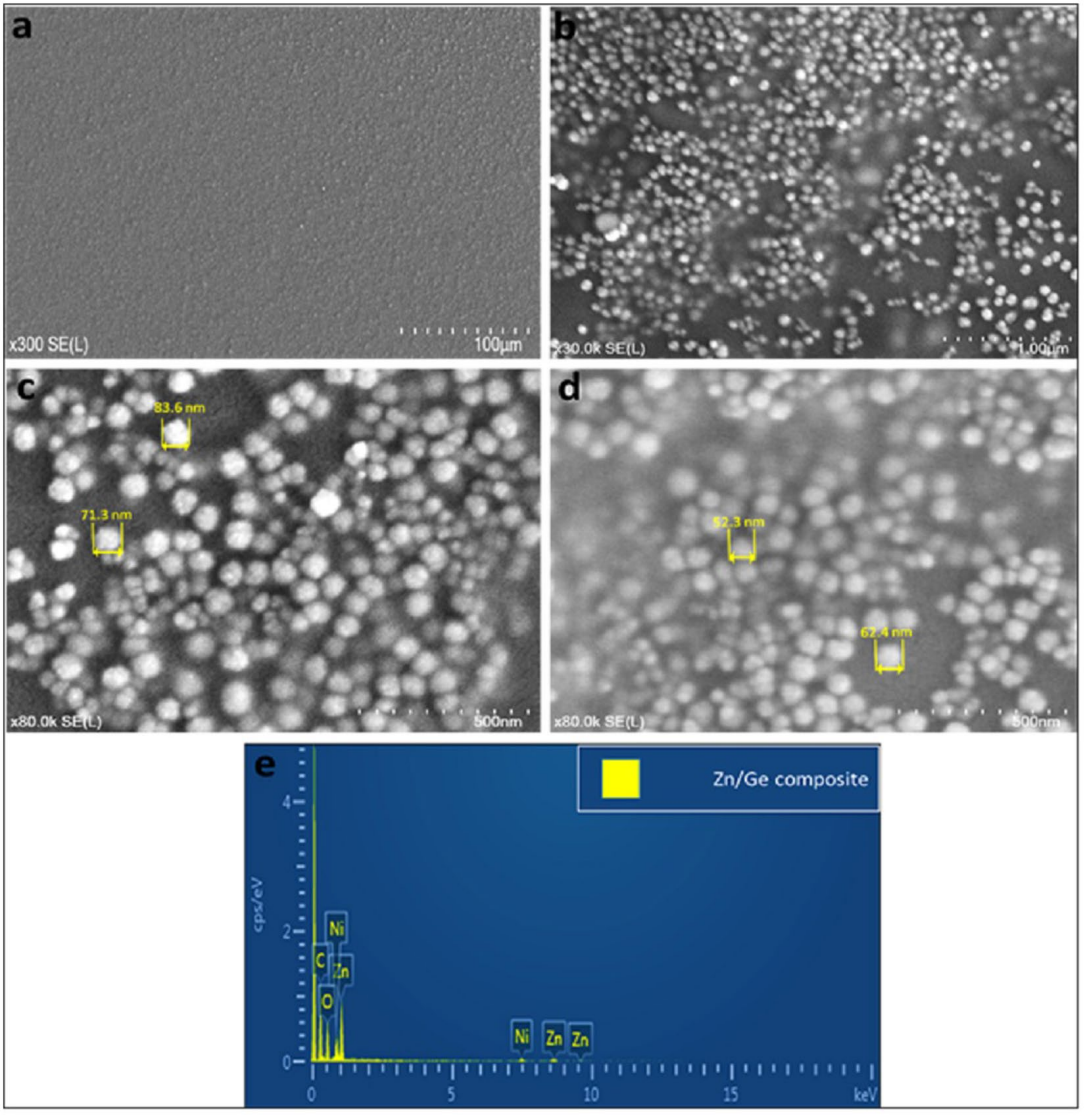
Typical SEM images of Zn/Ge 10 W composites at different magnifications, (**a**) is x300; and (**b**) is x30K. SEM images of (**c**,**d**) represent Zn/Ge 50 W and Zn/Ge 10 W, respectively; (**e**) shows EDS spectrum of the composite fabricated on Nickle substrates. SEM images were acquired at conditions of: Vacc = 3.0 kV, EC = 115 K nA, and WD = 6.1 mm.

Typical scanning electron microscope (SEM) images were obtained to determine the size and morphology of the formed nanoparticles in the polymer matrix, as seen in Fig. 4. It is known that at relatively high temperatures, typical of solid–vapor deposition methods, the growth conditions play a crucial role in the properties of resultant nanoparticles, where the morphology, shape and size could be significantly varied by altering parameters of the process^35,36^. The growth conditions of this study created ball-like nanoparticles that were clearly seen in all composites. ZnO particles were evenly distributed in the polymer medium. No significant morphological differences were observed between composites fabricated at 10 W and 50 W. However, the size of ZnO particles slightly increased with an increasing power of deposition. The average particle size increased with the input power, from approximately 60 nm at 10 W to approximately 80 nm at 50 W. The increase in particle size is in agreement with other studies on plasma polymers and metallic nanoparticles^37,38^. This indicates that a greater quantity of zinc can be incorporated into the polymer films at higher powers of deposition, agreeing well with the XPS data.

The cohesive energy of metals is typically higher (by two orders of magnitude) than the cohesive energy of polymers, which promotes a tendency for physical aggregations of metal atoms present within polymers^39^. These aggregations further increase by the very weak interaction between metals and polymers compared to strong metal–metal binding forces^1^. When active metal atoms are inserted and attach to the polymer, they go through various events, such as diffusion into the bulk, random rolling on the surface, or desorption. In the process of their diffusion across the polymer surface, metal atoms could be captured by surface cracks or encounter other metal atoms or particles, thereby forming aggregations and stable metal clusters. These accumulations are presented in the polymer structure during formation of the nanocomposite film. Here, we observed some of these unavoidable particle aggregations, representing less than 10% of the total number of nanoparticles. The EDS data in Fig. 4(e) further confirmed the presence of zinc in all composites, at peaks of 1.01, 8.63, and 9.57 keV. Peaks attributed to carbon and oxygen appeared at 0.27 and 0.52 keV, respectively. Nickel peaks were detected at 0.84 and 7.48 keV, representing the underlying substrate.

Thermal decomposition of zinc acetylacetonate hydrate (Zn (C_5_H_7_O_2_)_2_ ·xH_2_O) was also reported to form metallic zinc^30^, and zinc oxide^40,41^. The quantity, chemical state, particle size, and morphology of the formed zinc nanoparticles could vary considerably depending on the fabrication conditions^42^. However, this decomposition approach has a complex mechanism, with the bulk of transformations taking place when the material is heated above 130 °C. Indeed, the presence of H_2_O molecules in the Zn(acac)_2_ structure makes the breakdown process more complicated^43^. Water probably enhances the thermal decomposition by attacking oxygen of the carbonyl groups in acetylacetonate, and/or starting a direct reaction with zinc^43^. Arii *et al*. suggested that the thermal process is initiated with a single-step dehydration at ~110 °C, followed by complex sequential and parallel reactions, including phase transition, fusion, evaporation, and breakdown of anhydrous zinc acetylacetonate. The following formula describes thermal decomposition of acetylacetonate hydrate^44^:

2 $$Zn{(C{H}_{3}COCHCOC{H}_{3})}_{2}.{H}_{2}O\to ZnO+2C{H}_{3}COC{H}_{2}COC{H}_{3}$$

#### Atomic force microscopy

**Figure 5:**
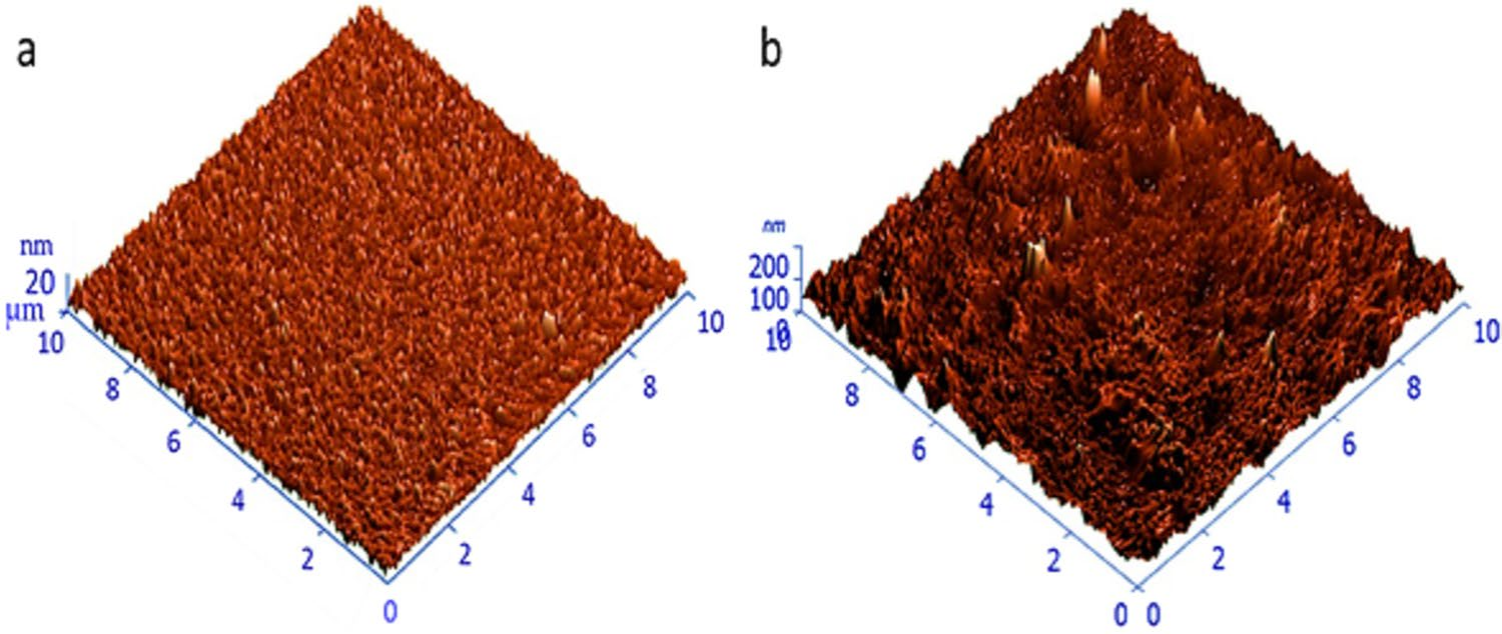
Three-dimensional AFM image of (**a**) pristine geranium polymer films, and (**b**) zinc/composites films. The scanning area of both images is 10 µm × 10 µm.

The surface morphology of the pristine and composite films was inspected by atomic force microscopy (AFM) using tapping mode atomic force microscopy (due to soft geranium polymer surface), with the results summarized in Table 2. As can be clearly observed in Fig. 5(a), the surface of the pristine polymer was smooth and uniform, with an average particle roughness of around 0.25 nm. As might be expected, the average roughness parameter significantly increased upon incorporation of zinc material, measuring 33.7 ± 2.1 and 37.2 ± 2.4 nm for 10 W and 50 W, respectively. The rather high surface roughness in Fig. 5(b) in comparison with the pristine film indicates that the composite exposes a porous surface with random distribution of ZnO particles. The symmetry of the deviations of a surface profile can be well-defined by using the surface skewness parameter, since it is sensitive to the irregularity of high peaks or deep grooves. A clear increase in the values of the surface skewness can be observed in composite films compared to pristine films. This probably relates to the presence of zinc nanoparticles on the surfaces of the films in the form of protrusions. To describe the distribution of these protrusions with respect to the mean line, a surface kurtosis value can be employed. For an identical protrusion distribution, the kurtosis is zero. The positive values of kurtosis indicates a high peak-type distribution (leptokurtic), while negative kurtosis refers to a flat-topped distribution (platykurtic). All composites showed high positive kurtosis values (coefficient of kurtosis ~2) indicating that the surfaces were dominated by high peaks.

**Table 2:** Surface profiles of 10 µm × 10 µm of pristine geranium film surfaces and zinc/composite films fabricated at various 10 W and 50 W powers.

Sample	Ge 10 W	Ge 50 W	Zn/Ge 10 W	Zn/Ge 50 W
Max, (nm)	3.75	3.52	363.3	385.1
Average Roughness, (nm)	0.23	0.30	33.7	37.2
Root Mean Square, (nm)	0.30	0.38	45.5	44.7
Surface skewness,	0.08	0.04	1.00	0.94
Coefficient of kurtosis	0.55	0.03	2.12	2.04
Entropy	3.45	3.80	1.54	2.70

#### Water contact angle

**Figure 6:**
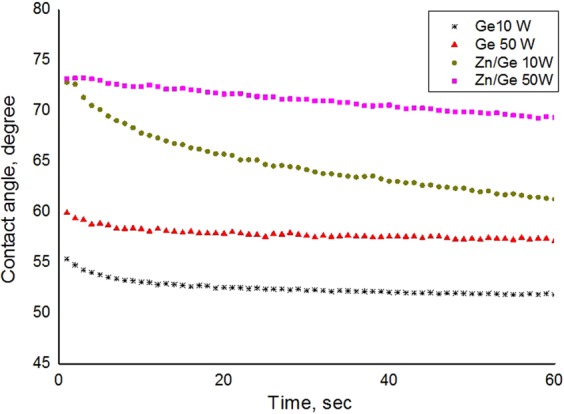
Water contact angle values for pristine and zinc/composite films fabricated at 10 W and 50 W. The data represent means of five replicates, with standard deviations of ±4° for the entire data set.

Wettability of the polymeric surface is an essential property that is mainly determined by the chemical composition and morphological structure of the surface. The static water contact angles of pristine and composite geranium films are shown in Fig. 6. An increase in the main contact angle from 54.0° to 61.2° can be observed for pristine samples fabricated in 10 W and 50 W respectively.

In the case of pristine polymers, plasma fabrication at higher input power conditions produces polymers with highly crosslinked structures due to more fragmentation/dissociation of precursor molecules. This results in the formation of relatively more-rigid polymers as a result of an increase in the bonding interconnection and dense packing of polymer chains within the matrix, minimizing the absorption of water on the surface. This may lead to an increase in the contact angle value, verifying the dependence of contact angle on the degree of crosslinking. Moreover, the increase in the input power affects the surface chemistry of the polymers, specifically a decrease in the oxygen content, resulting in a decrease in the polarity of the polymers’ surfaces. XPS data showed a reduction in oxygen quantity in the films produced at a higher RF power. Studies have reported an increase in the contact angle values when the input power was increased^45,46^.

In the case of composite Zn/Ge films, samples fabricated at 50 W revealed higher contact angle values, at approximately 73.4°, compared to contact angle values of approximately 66.4° from samples fabricated at 10 W. This is probably related to the difference in the size of formed nanoparticles at a different power of deposition. It had previously been reported that contact angles of ZnO nanoparticles are higher for larger particle sizes and lower as the particle size is reduced^47^. This agreed well with our findings, where composites fabricated at 50 W comprised larger nanoparticles, while composites fabricated at 10 W showed nanoparticles of smaller sizes. Furthermore, an increase in the contact angle can be understood in terms of a difference in the surface roughness parameters. As is known, nanoparticles deposited in a vacuum consist of a number of small crystallites, which contain a number of nano- and micro-scale air pockets. The impact of these air pockets can be cumulative once they interface with the droplet of liquid, efficiently supporting the weight of the droplet and effectively increasing the macroscopic contact angle. According to the Cassie-Baxter theory, the higher the section of the area of air that is under the droplet, the higher the value of the contact angle^48^. Our results agree with other studies that reported an increase in the contact angle when ZnO nanoparticles were incorporated into polymers^49–51^.

### *In vitro* antimicrobial performance

**Figure 7:**
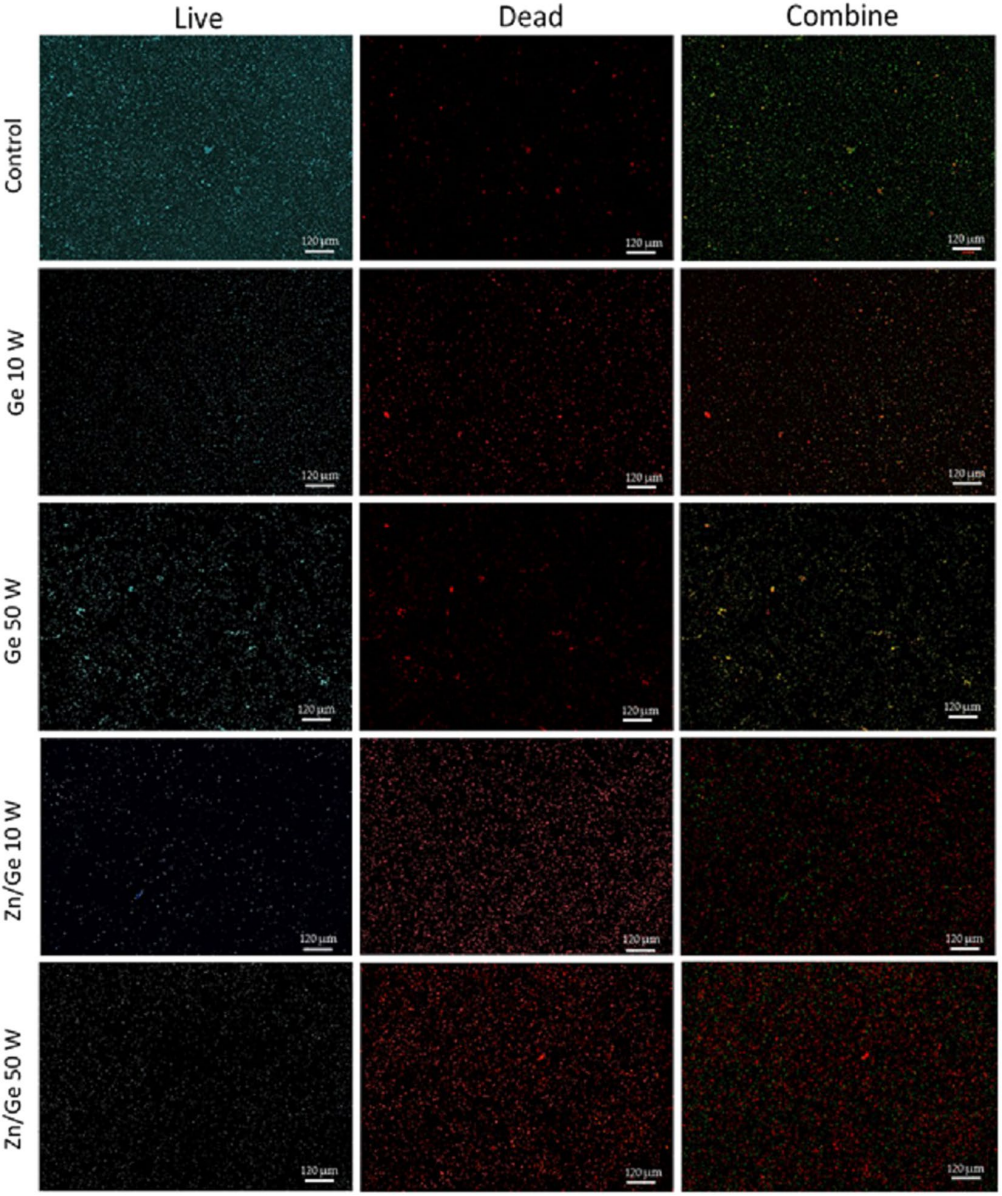
Confocal scanning laser microscopy images of *S. aureus* on control, Ge 10 W, Ge 50 W, Zn/Ge 10 W and Zn/Ge 50 W visualize viable cells stained green and dead cells stained red with Invitrogen Dead/Live Kit, with their combined images.

**Figure 8:**
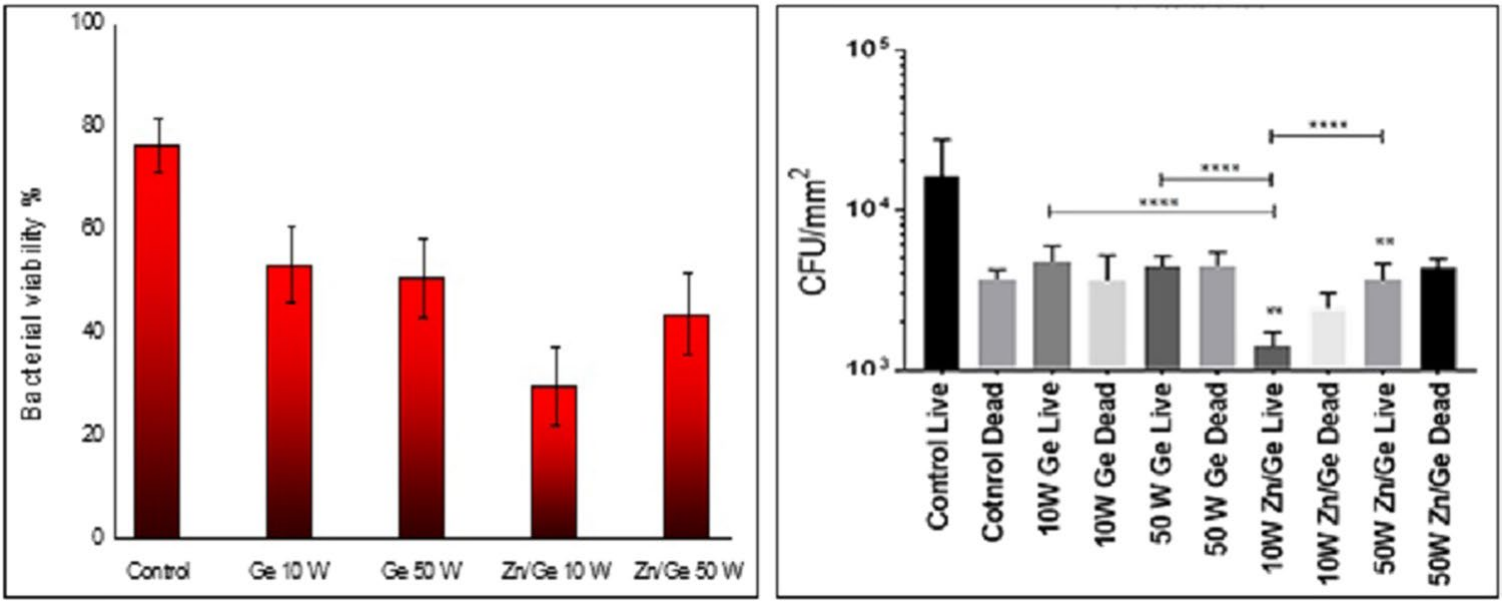
The bar chart (left) shows *S. aureus* viability on the samples. The statistical analysis chart (right) displays the antibacterial performance of all samples. Data shown represent means ± SD (n = 3). The statistical significance is given in terms of *(P < 0.05).

**Figure 9:**
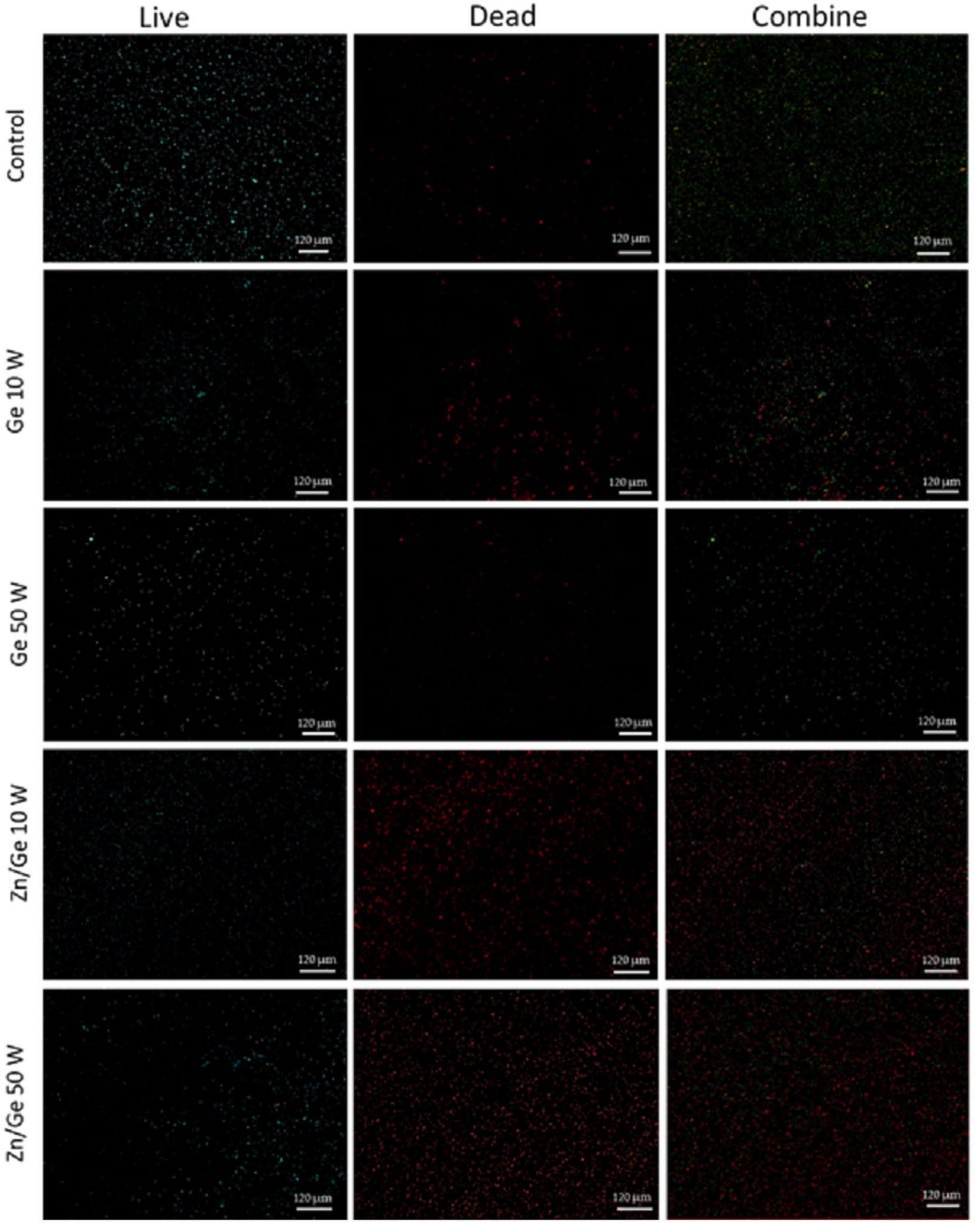
Confocal scanning laser microscopy images of *E.coli* on control, Ge 10 W, Ge 50 W, Zn/Ge 10 W and Zn/Ge 50 W visualize viable cells stained green and dead cells stained red with the Invitrogen Dead/Live Kit.

**Figure 10:**
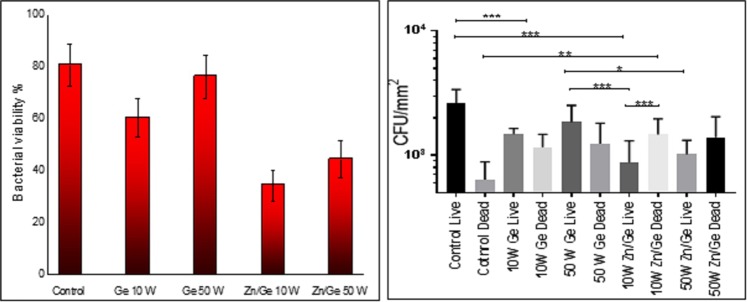
The bar chart (left) shows the bacterial viability on the samples. The statistical analysis chart (right) displays the antibacterial performance of all samples. Data shown represent means ± SD (n = 3). The statistical significance is given in terms of *(P < 0.05).

Figure 7 shows the bacterial viability of gram-positive *S. aureus* on surfaces of control, Ge 10 W, Ge 50 W, Zn/Ge 10 W and Zn/Ge 50 W. The Confocal scanning laser microscopy (CLSM) imaging evidently showed a much higher average number of cells adhered to the control surface compared to all polymers and composites samples. Figure 8 indicates that approximately, 80% of *S. aureus* were alive on the control, while the viability of cells were 53%, 50%, 31% and 42% on Ge 10 W, Ge 50 W, Zn/Ge 10 W and Zn/Ge 50 W, respectively. Similarly, Fig. 9 displays the bacterial viability of gram-negative *E. coli* cells on the control, pristine and composite films. On the control, 81% of *E. coli* were viable on the surface, while the viability of cells were approximately 60%, 76%, 33% and 44% on Ge 10 W, Ge 50 W, Zn/Ge 10 W and Zn/Ge 50 W, respectively, as seen in Fig. 10.

The bio-activity of essential oils is often linked to the chemical structure of its constituent molecules, mainly to functionalities that govern solubility in water and lipophilicity, as these characteristics allow these molecules to approach, disturb and pass through a bacterial membrane^52^. These disruptions then initiate membrane expansion, increase membrane permeability, induce disorder of membrane embedded proteins, inhibit respiration, and impact ion transport processes in the microbial cell. For example, carvacrol (a monoterpenic phenol) was found to make the cell membrane permeable to K+ and H+, dissipating the proton motive force and reducing ATP production^53^. Despite the fact the relation between chemical structure and bio-activity is not fully understood, it is generally accepted that oil constituents with a –OH group are more biologically effective than their similarly-structured OH-lacking counterparts^31,54^. However, when introducing essential oil molecules into plasma-created oxygen species, light and heat leads to their isomerization, oxidation, polymerization, dehydrogenation and thermal re-arrangements^4^. This results in the formation of a wide variety of biologically-active derivative monomers, dimers and polymers, reactive oxygen species and a wide range of oxidation ions. The degree of molecule dissociation is highly reliant on the processing parameters of the applied plasma. Thus, created oil-fragments then undergo recombination in the gas phase and on the surface of the given substrate, resulting in a highly crosslinked polymer with a structure that is irregular. Perhaps, plasma would weaken/deteriorate the antimicrobial activity of the original oil, as a lot of molecules would be cross-linked and as such, unable to interact with cellular membranes^52^.

The antibacterial performance of pristine polymers derived from essential oils is largely associated with the surface chemistry and nanoscale topography of the films^55^. The cellular and enzymatic activities of the adhered bacteria could be inhibited by the presence of bio-active functional groups (e.g. hydroxyl, carboxylic, methyl), disturbing the microbial growth and/or preventing a proper attachment of microbial cells^46^.

Although pristine polymers showed weak or moderate antibacterial activity against both gram-positive and gram-negative microorganisms, composites revealed enhanced bactericidal performance. It is obvious that zinc nanoparticles were directly involved in the inhibition of the pathogens. As shown in Figs 7 and 9, the antibacterial performance of Zn/Ge 10 W was greater compared to Zn/Ge 50 W. This observation can be linked to the influence of RF power on the size of the formed nanoparticles, where lower power created smaller particles. It is known that in many cases, smaller nanoparticles reveal much higher antibacterial action in compare to larger nanoparticles under the same conditions^56,57^.

**Figure 11:**
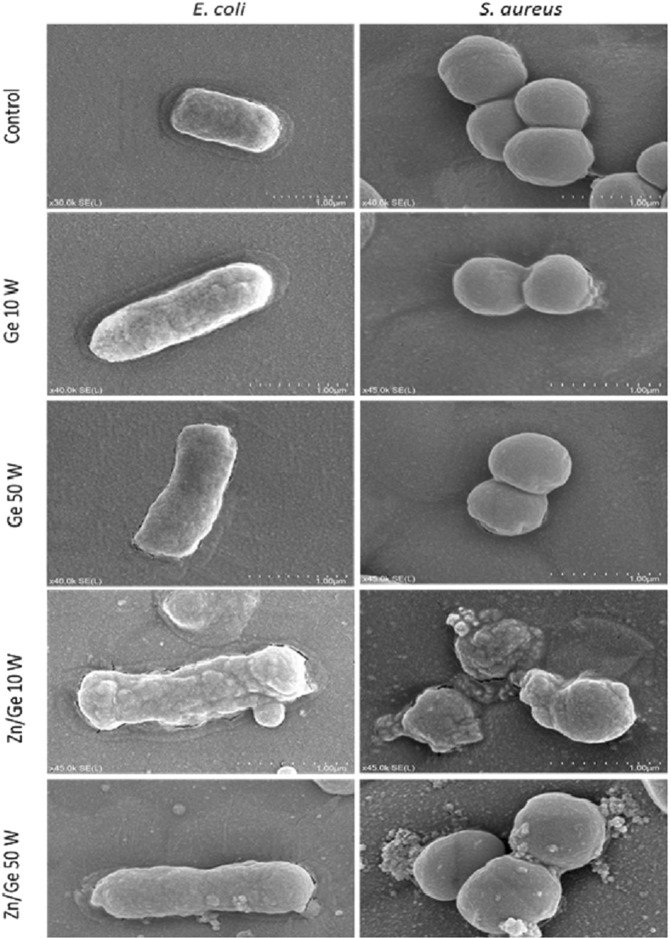
Morphologic observations of *E. coli* and *S. aureus* incubated for 24 h on surfaces of glass (control), Ge 10 W, Ge 50 W, Zn/Ge 10 W and Zn/Ge 50 W. SEM images were acquired at Vacc = 5.0 kV and WD = 7.5 mm.

Figure 11 displays SEM images of *S. aureus* and *E. coli* on the surfaces of control, Ge 10 W, Ge 50 W, Zn/Ge 10 W and Zn/Ge 50 W samples. SEM images show that bacteria incubated on Zn/Ge 10 W and Zn/Ge 50 W were clearly damaged and had disorganized cell walls, comparted to pristine polymers and control. The morphological changes were confirmed in both microorganisms upon interaction with ZnO NPs. Untreated bacteria displayed membrane structure integrity with a smooth surface. In contrast, the surface of bacteria appeared pimply and disorganized, indicating that ZnO NPs might damage and penetrate the membrane of the bacterial cell. The antimicrobial mechanisms of metallic nanoparticles are principally different from those of pristine polymers, providing further strategies for targeting various microorganisms, as well as to decrease the possible microbial resistance. Physicochemical changes often take place in cell membranes when nanoparticles anchor to the outer surface of the bacterial body, causing large variation in the permeability of cell walls^58^. Nanoparticles are also capable of entering, in a distinct way, the cell, due to the ultra-small size of these particles. The precipitation of NPs gather in the cytoplasm, or in the periplasm space, disrupting cellular activities, causing membranes disturbance and disorder. Brayner *et al*. reported that the interaction between *E. coli* and ZnO-NPs led to significant damage to the bacteria (e.g. disordered cell walls) and penetration of NPs into the cell, followed by intracellular content leakage^59^. Furthermore, upon interaction with bacterial cells, ZnO may generate reactive oxygen species (ROS), such as hydrogen peroxide (H_2_O_2_), hydroxyl radicals (OH^•^), superoxide radicals (O^−^_2_), and singlet oxygen (O^∗^_2_). At a high rate, these chemically-reactive species can be ruthlessly damaging to a bacterial cell owing to deterioration of proteins, peptidoglycan, ribosomes, DNA, as well as inhibition of enzymatic activities and amino acid production, ultimately leading to cell lysis^60,61^. So, we believe that Zn/Ge 10 W samples have shown higher performance as a result of a combination of different bactericidal mechanisms.

Many reports have shown that gram-positive bacteria appear to be more resistant against ZnO NPs^62,63^. This is linked to the specific thick and negatively charged peptidoglycan layer in the bacterial cell wall that can resist ZnO penetration. In contrast, other findings have found that gram-negative bacteria are more resistant to NPs activities^64–66^. Further, ZnO NPs have been reported to possess almost similar bacterial toxicity towards both microbial species^67^. There is an evident lack of consensus around the susceptibility of different bacteria to ZnO, possibly due to differences in experiments. Indeed, ZnO NPs are currently manufactured with different dimensions, morphologies, concentrations, surface modifications, surface defects, surface charges, crystallographic orientation, etc. The antibacterial mechanisms vary based on the physicochemical properties of nanoparticles and their microenvironment conditions. For example, in different media, the dissolving profile of Zn may vary according to the medium components, and accordingly, potentially influence the follow-on toxicity mechanism^68^. However, in our experimental conditions, attached bacterial cells on the Zn/Ge composites were not exposed solely to ZnO nanoparticles, rather, they were simultaneously affected by both the released ZnO and geranium polymer surfaces. Geranium polymers contain chemical groups (e.g. hydroxyl and carboxyl), which were proven in a previous study to reveal moderate antibacterial and anti-biofouling activities^45^. The inhibitory effects of Zn/Ge composite films were observed to be very similar on *S. aureus* and *E. coli*. Furthermore, we expect that interactions between the geranium oil vapors (contain alcohols) and ZnO NPs occurred during the fabrication of these composites. Here, more desired functional groups are presented on the ZnO surface, which may modify its biological activity. Surface modifications of the ZnO NPs in oxidizing environments had been demonstrated to cause an important change in the antibacterial activity^69^, which can be utilized in biomaterials. For example, Galindo *et al*. modified the surface of ZnO NPs by chemical components including di-functional alcohol, where these metallic particles can be used in antimicrobial medical devices^70^.

The antimicrobial performance of Zn/Ge composites is governed by a wide range of intrinsic factors. Given the close interconnection between fundamental polymer characteristics (e.g. surface chemistry, wettability, degree of cross-linking and topography) and NPs properties (e.g. shape, size, particle charge, concentration and crystallinity), it is possible that the synergistic outcomes of these factors inhibited bacterial viability. For example, Potara *et al*. synthezes bio-nanocomposites from silver nanoparticles and chitosan, which act synergistically against pathogenic bacteria^71^. Similarly, Prasad *et al*. found that nanocomposites (made from graphene and silver nanoparticles) are significantly more effective against pathogens than either individual components^72^. Furthermore, other factors, such as temperature, UV/light, pH, and species of the microorganism could influence the antibacterial performance of these composites^73,74^.

### Zinc release profile

**Figure 12:**
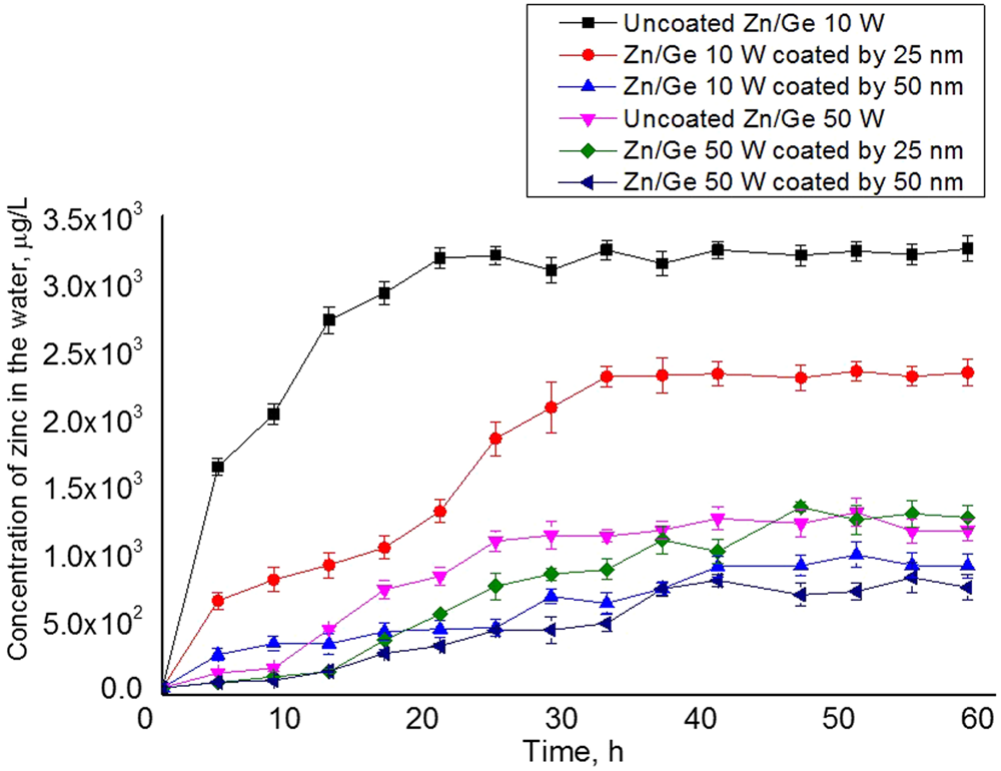
The concentration of the released-zinc in the water from Zn/Ge 10 W and Zn/Ge 50 W nanocomposite and its bi-layer system. The data were acquired in 5 ml of double distillated water in dark conditions using inductive coupled plasma measurements.

The release of zinc nanoparticles from Zn/Ge 10 W and Zn/Ge 50 W were confirmed using ICP measurements, and showed a relatively quick and sharp release profile of ZnO NPs within less than 24 h, as shown in Fig. 12. A high rate of nanoparticle release could be preferred in various medical applications. For example, a rapid release offers powerful antibacterial activity during the early post-operation period, inhibiting the possible development of microbial resistance^75^. Nevertheless, for other applications, such as implanted synthetic devices, surfaces should preserve their antibacterial performance until integration with the surrounding tissues, with sustained prevention of microbial colonization, and subsequent biofilms formation^76^. Thus, it is vital to adjust the releasing rate to match a specific application. In this regard, a recent approach to control the release of zinc oxide nanoparticles from coatings is to use a bi-layer polymeric system. Zn/Ge 10 W and Zn/Ge 50 W composites were coated by an extra thin layer (25 ± 5 and 50 ± 5 nm) of geranium film to reduce the releasing rate of zinc, which consequently increased the performance time. The measurements showed that the rate of release decreased, extending the time to 40 and 60 h for Zn/Ge 10 W and Zn/Ge 50 W composites, respectively. The slowest release rate was observed for composites coated with a 50 nm-think top layer.

A previous report showed that metallic nanoparticles (size of 15 nm and 70 nm) can pass through a thin upper layer of plasma polymer (thickness of 30 nm) upon immersion in aqueous solvents^77^. Another study demonstrated that increasing the thickness of the upper polymeric layer achieves a reduction in the rate of release of metal ions compared to composites without such an upper plasma film^78^. The same study proved that the upper layer coatings (thicknesses of 6, 12, and 18 nm) did not affect the antibacterial activities of the nanoparticles^78^. In our Zn/Ge composites, we observed a similar trend, as a thicker geranium upper layer would provide more robust polymers, further reducing the release rate of nanoparticles without major variations in the biological performance of the nanocomposites. This indicates that the rate of release can be adjusted via the thickness of the thin barrier plasma polymer. The upper layer polymeric layer may function as both a 3-D matrix for protection of the nanoparticles from the medium, and to delay the release of zinc, potentially yielding a variety of release profiles. Obviously, the thickness of the upper layer should be carefully selected to ensure the maximum release of the nanoparticles, where the bulk of nanoparticles could be trapped inside the upper layer; to be comprehensively investigated in an upcoming study.

ICP results in Fig. 12 show that composites fabricated at a higher input power (50 W) possess a considerably lower release rate compared to composites fabricated at 10 W. This can be linked to the degree of cross-linking in the material. It has been shown that fabricating geranium plasma polymers at higher power leads to an increase in the degree of cross-linking, and potentially rise resistance against deformation^45^. The resultant polymers become harder and more rigid owing to the increase in the bonding interconnection and dense chain packing^79^. Here, we assumed that the increase in the bonding interconnection degree would curb/restrain the freeing of nanoparticles from the films fabricated at 50 W. ICP data also suggested that a higher concentration of ZnO was released from surfaces of ZnO/Ge 10 W, making it significantly more antibacterially effective than ZnO/Ge 50 W that showed a low degree of release hence, much lower antibacterial activity.

In addition to the influence of the cross-linking degree, the size of nanoparticles could also contribute to the release profile of zinc. As shown by our SEM data, when the power of deposition increased, the average size of the ZnO nanoparticle slightly increased (from approximately 60 nm at 10 W to approximately 80 nm at 50 W). It is reasonable to assume that a smaller size particle has higher mobility in the polymer and delivers a further swelling rate, compared to a larger-sized counterpart. Consequently, the particle will be easily released from the polymer matrix in the aqueous solution. Yet, it was reported that the major contributors of a controlled release of ZnO from a film are related to the host properties, mainly the degree of intermolecular interactions rather than NPs’ morphological features such as particle shape and size^80^.

## Conclusion

We successfully developed a nanocomposite material from geranium essential oil and the thermal decomposition of zinc acetylacetonate in a one step plasma system. XPS survey confirmed the presence of ZnO in the fabricated polymers at 10 W and 50 W. SEM images showed that the average size of the ZnO nanoparticle increased with an increase in the power of deposition, from approximately 60 nm at 10 W to approximately 80 nm at 50 W. AFM data showed a significant increase in surface roughness of the composites compared to pristine samples. The antibacterial activity of ZnO-NPs incorporated into geranium films toward both gram-positive (*S. aureus*) and gram-negative (*E. coli*) bacteria were demonstrated. The release of nanoparticles was confirmed using ICP measurements, and can be further controlled through a bi-layer system. These coatings were confirmed to be a promising candidate for protecting relevant medical devices and implants’ material surface from bacterial attachment and colonization.
